# Isolation of Fission Yeast Condensin Temperature-Sensitive Mutants with Single Amino Acid Substitutions Targeted to Hinge Domain

**DOI:** 10.1534/g3.119.400156

**Published:** 2019-03-26

**Authors:** Xingya Xu, Mitsuhiro Yanagida

**Affiliations:** G0 Cell Unit, Okinawa Institute of Science and Technology Graduate University, Onna-son, Okinawa 904-0495, Japan

**Keywords:** site-directed mutagenesis, temperature-sensitive mutant, cold-sensitive mutant, condensin, hinge

## Abstract

Essential genes cannot be deleted from the genome; therefore, temperature-sensitive (ts) mutants and cold-sensitive (cs) mutants are very useful to discover functions of essential genes in model organisms such as *Schizosaccharomyces pombe* and *Saccharomyces cerevisiae*. To isolate ts/cs mutants for essential genes of interest, error-prone mutagenesis (or random mutagenesis) coupled with *in vitro* selection has been widely used. However, this method often introduces multiple silent mutations, in addition to the mutation responsible for ts/cs, with the result that one cannot discern which mutation is responsible for the ts/cs phenotype. In addition, the location of the responsible mutation introduced is random, whereas it is preferable to isolate ts/cs mutants with single amino acid substitutions, located in a targeted motif or domain of the protein of interest. To solve these problems, we have developed a method to isolate ts/cs mutants with single amino acid substitutions in targeted regions using site-directed mutagenesis. This method takes advantage of the empirical fact that single amino acid substitutions (L/S -> P or G/A -> E/D) often cause ts or cs. Application of the method to condensin and cohesin hinge domains was successful: ∼20% of the selected single amino acid substitutions turned out to be ts or cs. This method is versatile in fission yeast and is expected to be broadly applicable to isolate ts/cs mutants with single amino acid substitutions in targeted regions of essential genes. 11 condensin hinge ts mutants were isolated using the method and their responsible mutations are broadly distributed in hinge domain. Characterization of these mutants will be very helpful to understand the function of hinge domain.

Forward genetic screens of ts/cs collections for mutants exhibiting specific phenotypes identified essential genes involved in cell division or chromosome segregation (Nurse and Thuriaux 1980; Hirano *et al.* 1986). Reverse genetic screens that analyzed ts/cs mutants of essential genes of interest have also been popular. To isolate ts/cs mutants for targeted essential genes, error-prone mutagenesis has been commonly used (Hayashi *et al.* 2014; Obuse *et al.* 2004; Xu *et al.* 2015). Typically error-prone PCR is performed under conditions (usually by increasing Mg^2+^ concentration in the reaction) to reduce the fidelity of DNA polymerase during DNA synthesis. The number of mutations increases with the number of gene duplication events (PCR cycles) (McCullum *et al.* 2010). If one uses higher Mg^2+^ concentration, ts or cs mutants are obtained more frequently after *in vitro* selection, but more mutations are observed in the ts or cs mutants. On the other hand, if one uses lower Mg^2+^ concentration, ts or cs mutants are obtained less frequently after *in vitro* selection, but fewer mutations are observed in the ts or cs mutants.

Proteins contain critical motifs or domains that execute specified functions. Condensin and cohesin are two related protein complexes essential for faithful chromosome segregation (Furuya *et al.* 1998; Hirano *et al.* 1986; Tatebayashi *et al.* 1998). All of their SMC (structural maintenance of chromosomes) subunits (Cut3 and Cut14 in condensin; Psm1 and Psm3 in cohesin) contain head and hinge domains that are separated by coiled coils (Hirano and Mitchison 1994; Strunnikov *et al.* 1993). Non-SMC subunits (Cnd1, Cnd2 and Cnd3 in condensin; Rad21, Psc3 and Mis4 in cohesin) bind to the head domains of SMC dimers. Several ts mutants are available for condensin and cohesin. For condensin, *cut14-Y1* (containing a L543S substitution in the hinge) (Akai *et al.* 2011), *cut14-aa14* (with T558L in the hinge) (Petrova *et al.* 2013), *cut14-208* (containing S861P in the coiled coil) (Sutani and Yanagida 1997), *cut3-477* (with S1147P in the coiled coil) (Sutani and Yanagida 1997) and *cnd2-1* (containing A114T in the N-terminal HTH motif) (Aono *et al.* 2002) were identified by screening for ts mutants exhibiting chromosome segregation defects. In addition, multiple *cnd* (*cnd1*, *cnd2* and *cnd3*) ts mutations for condensin non-SMC subunits were identified by error-prone mutagenesis (Xu *et al.* 2015). For cohesin, *rad21-K1* (having an I67F substitution in the N-terminal HTH motif) (Tatebayashi *et al.* 1998; Xu *et al.* 2018a), *psc3-407* (the responsible mutation is still unknown; Yuasa *et al.* 2004), and *mis4-242* (with a G1326E substitution) (Furuya *et al.* 1998) were identified by screening for ts mutants exhibiting chromosome segregation defects. In addition, 6 ts and 6 cs cohesin hinge mutants were isolated using the method described here, proving that this technique has considerable utility (Xu *et al.* 2018a).

Since the method worked very well to isolate ts/cs mutants with single amino acid substitutions in the cohesin hinge, we here describe the method in detail, and then apply it to target the condensin hinge, from which few mutants were available. ∼20% of the selected mutation sites turned out to be ts mutants, therefore the success of the method is not particular and it may be applicable to any targeted region of essential genes.

## Materials and Methods

### Strains, plasmids, and media

The wild-type strain 972 *h*^-^ was used as the host strain for ts mutant construction. ∼500bp long sequences (3′ UTRs) after the corresponding ORFs (cut3 or cut14) were cloned and ligated into pBluescript plasmids downstream of a hygromycin-resistance antibiotic marker (hygR). Complementary pairs of synthesized DNA oligos (∼35 bp) with designed mutations were used as PCR primers (Figure 2A, Table S1 and S2), followed by two rounds of PCR (Figure S1). Mutated genes (cut3 ORF or cut14 ORF) were cloned and ligated into the pBluescript plasmids (with 3′ UTR integrated) upstream of the antibiotic marker. Then the plasmids were linearized and chromosomally integrated into corresponding endogenous loci of the aforementioned 972 *h*^-^ wild-type strain using lithium acetate method described in Figure S2. Hygromycin-resistant colonies were selected on YPD plates containing 500μg/mL Hygromycin B (Wako), and then temperature-sensitive candidates, which can grow at 26° but not at 36°, were screened. ts mutations were confirmed by Sanger sequencing of mutated genes. YPD medium and plates (1% yeast extract, 2% polypeptone, 2% D-glucose) were used for culturing *S. pombe* strains (Forsburg and Rhind 2006).

### Protein alignment and visualization

Cut3 and Cut14 protein sequences were downloaded from Pombase (http://pombase.org) (Wood *et al.* 2012). Protein sequences of Cut3 and Cut14 homologs in other organisms were downloaded from the NCBI HomoloGene Database (https://www.ncbi.nlm.nih.gov/homologene) (Wheeler *et al.* 2001). Protein sequences were aligned using a multiple sequence alignment program MAFFT (https://mafft.cbrc.jp/alignment/software/) (Katoh *et al.* 2002). Protein alignment results were visualized using a multiple sequence alignment visualization tool, Jalview (http://www.jalview.org) (Waterhouse *et al.* 2009).

### Mutational analysis of condensin hinge ts mutations in 3D structures

An atomic model of the *S. pombe* condensin hinge (Akai *et al.* 2014) was generated from crystal structures of SMC hinges from *T. maritima* (PDB codes 1GXJ and 1GXL) (Haering *et al.* 2002) and mouse (PDB codes 2WD5 and 3L51) (Griese *et al.* 2010; Kurze *et al.* 2011) based on a sequence alignment using MODELER (Sali and Blundell 1993).

### Data availability

Strains are available upon request. The authors affirm that all data necessary for confirming the conclusions of the article are present within the article, figures, and the supplemental file. Table S1 and S2 contain lists the primers used to construct cut14 and cut3 mutants, respectively. Figure S1 describes PCR conditions used to introduce mutations into target genes. Figure S2 describes transformation protocol. Supplemental material available at Figshare: https://doi.org/10.25387/g3.7890911.

## Results

### Substitution of L/S -> P or G/A -> D/E often cause ts/cs

In previous studies, ts or cs mutants with specific phenotypes were isolated by forward genetic screening of the collection of ts/cs mutants (Nurse and Thuriaux 1980; Hirano *et al.* 1986). We selected and used those that exhibited chromosome segregation defects at the restrictive temperature. Many of them contain substitutions from Leucine (L)/Serine (S) to Proline (P) or from Glycine (G)/Alanine (A) to Aspartic Acid (D)/Glutamic Acid (E) (L/S -> P or G/A -> D/E), as shown in Table 1. In addition, in error-prone mutagenesis for *cnd* mutants, 10 of 21 ts or damage-sensitive mutants with single amino acid substitutions introduced proline (Xu *et al.* 2015).

**Table 1:** ts/cs mutants with amino acid substitutions L/S -> P or G/A -> E/D

Gene	Allele	ts/cs	aa change	Reference
cnd1	*cnd1-L193P*	ts	L193P	Xu *et al.* 2015
	*cnd1-L331P*	ts	L331P	
	*cnd1-L685P*	ts	L685P	
cnd2	*cnd2-ae9*	ts	L103P	Petrova *et al.* 2013
cnd3	*cnd3-L126P*	ts	L126P	Xu *et al.* 2015
	*cnd3-L269P*	ts	L269P	
cut3	*cut3-l23*	ts	S1116P	Petrova *et al.* 2013
	*cut3-477*	ts	S1147P	Sutani and Yanagida 1997
cut14	*cut14-208*	ts	S861P	
mis17	*mis17-362*	ts	S353P	Hayashi *et al.* 2004
cut8	*cut8-563*	ts	S201P	Takeda *et al.* 2011
mis14	*mis14-271*	ts	L106P	Hayashi *et al.* 2004
	*mis14-634*	ts	S130P	
nuf2	*nuf2-1*	ts	S189P	Nabetani *et al.* 2001
	*nuf2-3*	ts	L246P	
psm3	*psm3-A561E*	cs	A561E	Xu *et al.* 2018a
mis4	*mis4-242*	ts	G1326E	Furuya *et al.* 1998
mis6	*mis6-302*	ts	G135E	Saitoh *et al.* 1997
mis12	*mis12-537*	ts	G52E	Goshima *et al.* 2003
nda3	*nda3-KM311*	cs	G93E	Paluh *et al.* 2000
mis18	*mis18-262*	ts	G117D	Hayashi *et al.* 2004
nuc2	*nuc2-663*	ts	G504D	Samejima and Yanagida 1994
htb1	*htb1-72*	ts	G52D	Maruyama *et al.* 2006
eso1	*eso1-H17*	ts	G799D	Tanaka *et al.* 2000
cdc48	*cdc48-353*	ts	G338D	Ikai and Yanagida 2006
cut14	*cut14-r8*	ts	G10D	Petrova *et al.* 2013
clr6	*clr6-1*	ts	G269D	Grewal *et al.* 1998

### Selection of conserved L/S/G/A amino acids for mutagenesis

**Figure 1:**
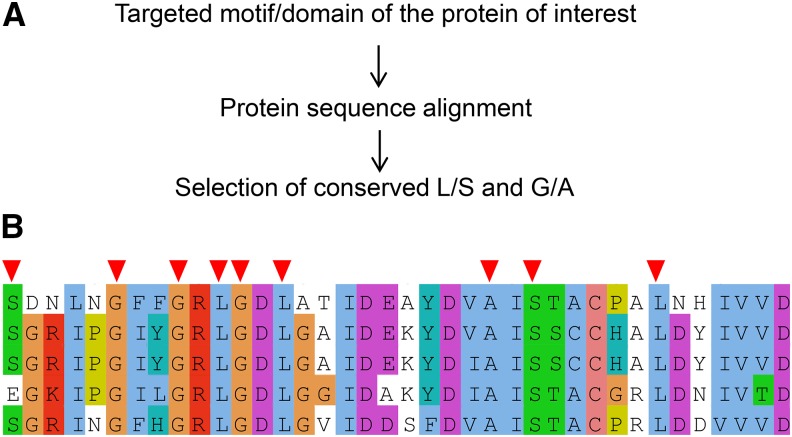
Selection of amino acids for mutagenesis. (A) A brief procedure to select conserved L/S/G/A amino acids in the motif/domain of interest in the target protein. (B) Protein alignment result of a portion of condensin Cut3 hinge as an example. Selected amino acids (L/S/G/A) for site-directed mutagenesis are marked with red arrowheads.

We intended to develop a ts/cs isolation method based on site-directed mutagenesis by taking advantage of the aforementioned observations (Table 1) to isolate ts/cs mutants with single amino acid substitutions in targeted motifs/domains of essential genes of interest. To perform site-directed mutagenesis, one needs to decide which protein and which domain/motif is the target based on available information (such as structural and functional annotations from a database for the fission yeast *Schizosaccharomyces pombe*, PomBase) (Wood *et al.* 2012). Then homologous protein sequences from different species are downloaded from databases, for example, the NCBI HomoloGene Database (https://www.ncbi.nlm.nih.gov/homologene) (Wheeler *et al.* 2001) and are aligned using MAFFT (https://mafft.cbrc.jp/alignment/software/) (Katoh *et al.* 2002). Based on alignment results, one needs to select conserved L/S/G/A amino acids in the target domain/motif (Figure 1A and 1B).

### Site-directed mutagenesis for mutants

**Figure 2:**
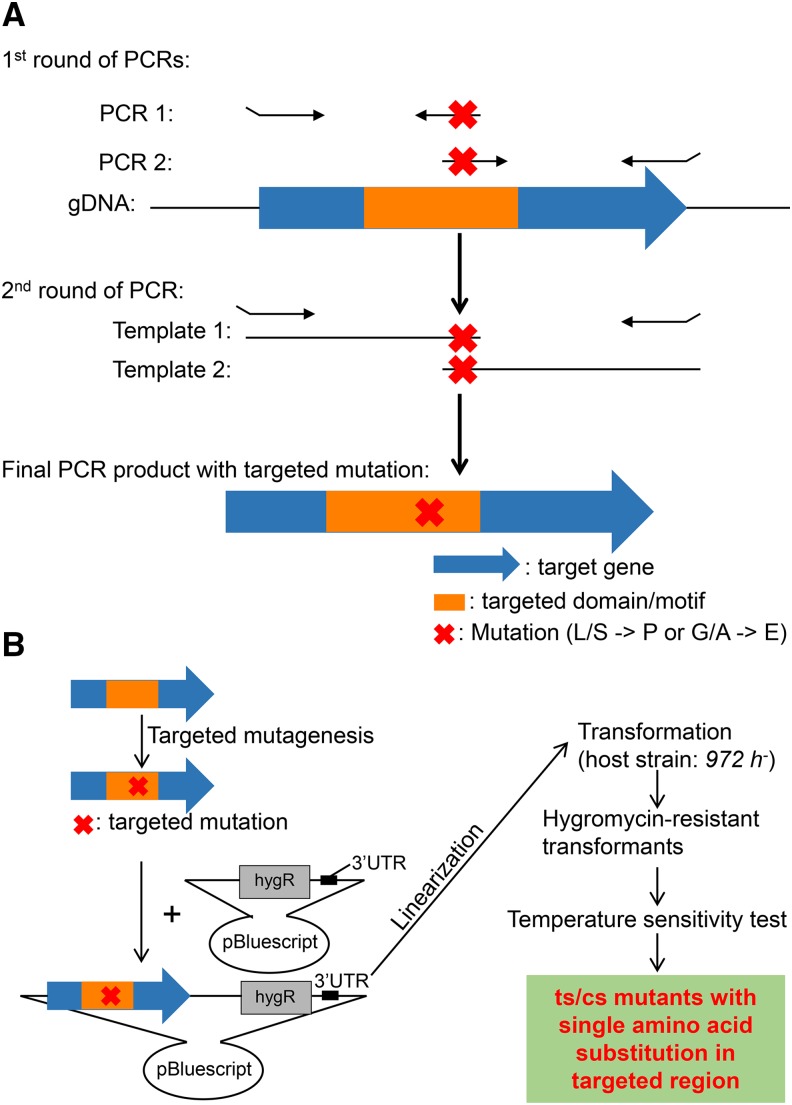
Site-directed mutagenesis. (A) Strategy to introduce targeted mutations into target genes using two-rounds of PCR. Primers used to introduce cut14 hinge mutations are presented in Table S1 and primers used to introduce cut3 hinge mutations are presented in Table S2. (B) Site-directed mutagenesis and isolation of ts/cs mutants. See ‘Materials and Methods’ for detailed description of plasmid construction and how to isolate ts/cs mutants.

Based on mutations depicted in Figure 1, PCR primers are designed to introduce mutations into the DNA sequence of the target gene (see ‘Materials and Methods’, Figure S1 and Figure S2). Two rounds of PCR are performed by following the procedure described in Figure 2A. DNA polymerase and PCR conditions are described in Figure S1. The target gene with a designed mutation was ligated into a pBluescript plasmid with its 3′ UTR (∼500 bp DNA sequences downstream of the gene) already integrated. Linearized plasmids were transformed into the 972 *h*^-^ wild-type strain using lithium acetate method described in Figure S2. After hygromycin B (500μg/mL) selection for integrants, colonies were selected and streaked under three different culture conditions to measure their temperature sensitivity (36° for ts mutants, 30° as a control, and 20° for cs mutants) (Figure 2B). The number of colonies needed varies, but usually between 4 and 16 is sufficient.

### Cohesin hinge ts/cs mutants isolated

Suppressors that overcome inactive separase/Cut1 or securin/Cut2 were identified and found to be located at cohesin interfaces, including the hinge (Xu *et al.* 2018a). To examine *cut1*’s suppression by cohesin hinge mutations and to further understand the underlying mechanism, more cohesin hinge mutants were conceived. Specifically, 59 conserved L/S/G/A amino acids in the cohesin hinge (26 in the Psm1 hinge and 33 in the Psm3 hinge) were selected for site-directed mutagenesis. After screening for ts/cs mutants, 12 ts/cs mutants (6 ts and 6 cs) were obtained (∼20% of the total selected amino acids).

### Isolation of condensin hinge ts mutants

**Figure 3:**
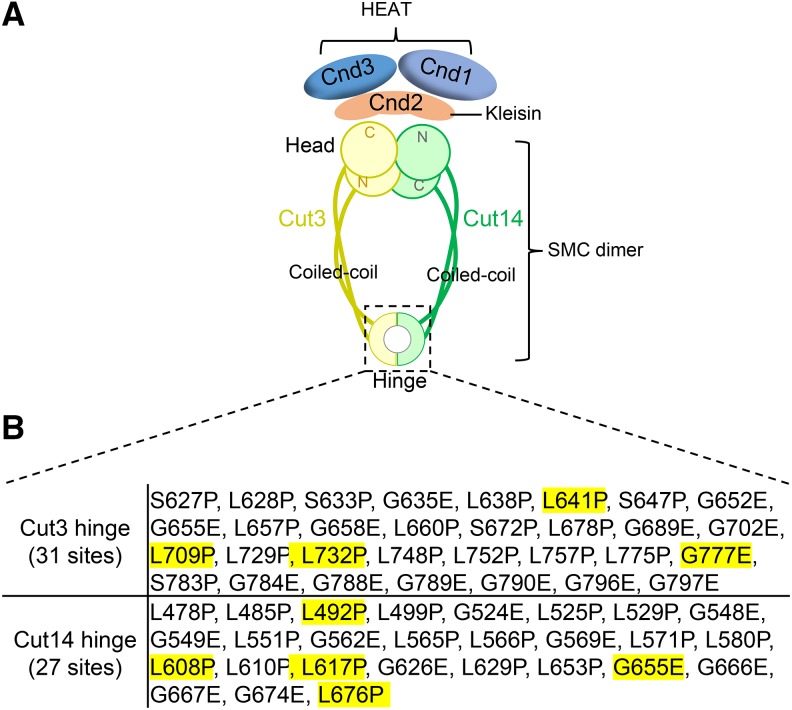
Mutations in the condensin hinge selected for site-directed mutagenesis. (A) Condensin is a heteropentameric complex required for faithful chromosome condensation and segregation. Cut3 and Cut14 hinges form a heterodimer containing two interfaces. (B) To understand condensin hinge’s function, conserved L/S/G/A amino acids were selected for site-directed mutagenesis. 31 mutations in Cut3 hinge and 27 mutations in Cut14 hinge were designed. Site-directed mutagenesis and isolation of condensin hinge ts mutants followed the procedure described in Figure 2B.

*cut14-Y1* (Akai *et al.* 2011) and *cut14-aa14* (Petrova *et al.* 2013) are the only two condensin hinge ts mutants containing L543S and T558L substitutions as their responsible mutations, respectively. No Cut3 hinge ts mutant is available yet. *cut14-Y1* was important to understand the function of the hinge in DNA association and release, and also led to the discovery of the hinge-head interaction through transient phosphorylation of the hinge by the head ATPase (Akai *et al.* 2011; Akai *et al.* 2014). Application of the method described here to isolate cohesin hinge ts/cs mutants was successful (Xu *et al.* 2018a); therefore, to examine the versatility of the method, here we applied the method to isolate condensin hinge ts mutants (Figure 3A). Based on the homologous protein sequence alignment, conserved L/S/G/A amino acids in the Cut3 and Cut14 hinge domains were selected. 31 mutations in the Cut3 hinge domain and 27 mutations in the Cut14 hinge domain were designed for site-directed mutagenesis (Figure 3B).

### Condensin hinge ts mutants obtained

**Figure 4:**
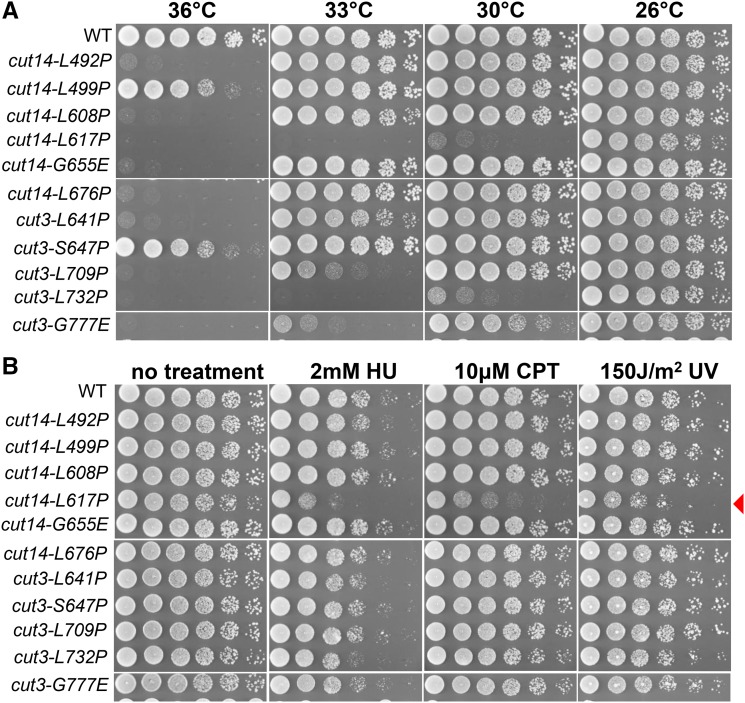
Condensin hinge ts mutants with single amino acid substitutions. (A) Spot test results describing the temperature sensitivity of the 11 condensin hinge ts mutants newly isolated from the 58 mutations described in Figure 3B. 9 of the 11 condensin hinge ts mutants are strongly sensitive to high temperature, while the temperature sensitivity of *cut14-L499P* and *cut3-S647P* is weak. (B) Sensitivity of newly isolated condensin hinge ts mutants to DNA damaging agents at the permissive temperature (26°C). Among the 11 ts mutants, only *cut14-L617P* is sentive to damage. HU, CPT and UV designate hydroxyurea, camptothecin and ultraviolet respectively.

In total, 11 ts mutants with single amino acid substitutions in condensin hinge domains were obtained and 9 of them presented strong temperature sensitivity (Figure 4A). In total, 58 conserved L/S/G/A amino acids were selected (Figure 3B); therefore, ∼20% of the single amino acid substitutions caused ts. If selection for cs mutants was considered too, perhaps more than 20% of the single amino acid substitutions cause ts/cs. Among the 11 newly isolated condensin hinge ts mutants, 6 are cut14 hinge ts mutants and the other 5 are cut3 hinge ts mutants. Since the previously isolated condensin hinge ts mutant, *cut14-Y1*, was hyper-sensitive to DNA damaging agents at the permissive temperature (26°) (Akai *et al.* 2011), we examined sensitivity of these newly isolated condensin hinge ts mutants to DNA damaging agents (hydroxyurea, camptothecin and ultraviolet) at the permissive temperature (26°). Among the 11 ts mutants, only *cut14-L617P* was sensitive to DNA damaging agents (Figure 4B). Therefore in this study, we identified 10 condensin hinge ts mutants, which are ts, but not sensitive to DNA damaging agents (at least at 26°. It’s still possible that some of them will be sensitive to DNA damaging agents when incubated at 30° or 33°).

### Localization of condensin hinge ts mutations

**Figure 5:**
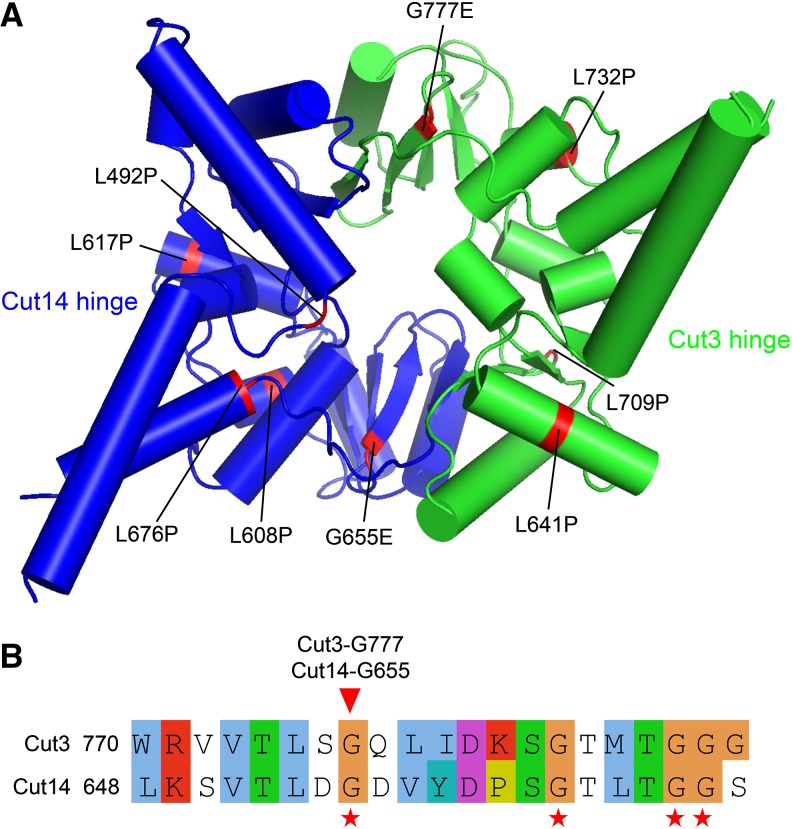
Localization of newly isolated condensin hinge ts mutations in the 3D structure. (A) Mutations of the newly isolated condensin hinge ts mutants were mapped onto the atomic structure of the condensin hinge. The Cut14 hinge is in blue and the Cut3 hinge is in green. The broadly distributed mutations are highlighted in red. (B) Alignment of the Cut3 and Cut14 hinges around the conserved arrangement of glycine residues (GX6GX3GG sequence motif), which is normally found in hinge dimerization interfaces. Conserved glycines are marked with red asterisks below the alignment. Localization of Cut14-G655 and Cut3-G777 is indicated with the red arrowhead. Cut14-G655 and Cut3-G777 are in same position, but at different interfaces.

To understand these ts mutations, we mapped them onto the condensin hinge structure and found that they are broadly distributed (Figure 5A). However, Cut14-G655 and Cut3-G777 are located at the two hinge dimerization interfaces. Protein sequence alignment between the Cut14 and Cut3 hinges indicated that Cut14-G655 and Cut3-G777 are at the same position in the 3D structure, but at different interfaces (Figure 5B). A conserved arrangement of glycine residues (GX6GX3GG sequence motif) is normally found in hinge dimerization interfaces (Figure 5B), and it is required for hinge dimerization (Hirano *et al.* 2001; Hirano and Hirano 2002; Hirano and Hirano 2006). Cut14-G655 and Cut3-G777 are located in this GX6GX3GG sequence motif. Mutation of Cut14-G655 or Cut3-G777 to E may affect hinge dimerization. Alternatively, a ‘hold and release’ model, in which head and hinge interact to form arched coiled coils that hold chromosomal DNAs inside, has been proposed (Xu *et al.* 2018a), Cut14-G655E and Cut3-G777E may affect the angle formed by the coiled coils emerging from hinge and further affect hinge’s DNA binding ability.

## Discussion

The method developed here can be used to isolate ts/cs mutants with single amino acid substitutions in targeted regions of essential genes in both *Schizosaccharomyces pombe* and *Saccharomyces cerevisiae*. It has great advantages in that single amino acid substitutions are introduced and mutations are located in the domain/motif of interest. ∼20% of conserved L/S/G/A amino acids selected for site-directed mutagenesis caused ts/cs; therefore, the chance to obtain ts/cs mutants is high using the method described here.

Error-prone mutagenesis (or random mutagenesis) was frequently used to isolate ts or cs mutants for essential genes. In a previous study using error-prone mutagenesis for ts mutants of condensin non-SMC genes (cnd1, cnd2 and cnd3) (Xu *et al.* 2015), in total 59 ts mutants were isolated from ∼18,000 transformant colonies (∼0.3%), and among these 59 ts mutants only 16 of them contain a single amino acid substitution (∼0.1%). In another study using error-prone mutagenesis for ts mutants of sam1 gene (which encodes a S-adenosylmethionine synthetase; Hayashi *et al.* 2018), 5 ts mutants were obtained from ∼3000 colonies screened (∼0.2%) and 2 of them contain a single amino acid substitution (∼0.07%). Therefore the frequency to get ts mutants with single amino acid substitution is low. By using the method described here, ∼20% of the designed mutations caused ts or cs, therefore there’s no need to screen many colonies. In addition, responsible mutations of the ts mutants, isolated using error-prone mutagenesis, is random (Hayashi *et al.* 2018; Xu *et al.* 2015), they may not locate in the domain or motif of interest. The method described here designs mutations, by taking advantage of the empirical fact that single amino acid substitutions (L/S -> P or G/A -> E/D) often cause ts or cs, in the target domain or motif of interest, therefore all the responsible mutations of the ts or cs mutants isolated are located in the target domain or motif. However, not much information about the genes of interest are required before error-prone mutagenesis, protein features of the genes of interest are required in this method and more plasmid construction works are required prior to screening.

The 11 ts mutants isolated for condensin hinge is valuable to understand hinge’s function. Among the 11 ts mutants, *cut14-L617P* and *cut3-L732P* are the strongest, they can’t grow even at 30° (Figure 4A). However, we still don’t know why their temperature sensitivities are the strongest only from the mutations’ locations in the hinge structure (Figure 5A). In cohesin, it is proposed in a ‘hold and release’ model (Xu *et al.* 2018a) that hinge mutations affect coiled coils that are connected to hinge. Orientation of coiled coils, which hold and release chromosomal DNAs in between, was largely changed in a cohesin hinge cs mutant, *psm3-A561E*. Whether these condensin ts mutants affect coiled coils connected to the hinge is still unknown yet.

An efficient and cost-effective suppressor mutation identification method using next-generation sequencing of genomic DNA Mixture was developed (Xu *et al.* 2018b). Therefore, ts/cs mutants isolated can be further applied for suppressor screening. The combination provides a complete pipeline to understand the function of essential genes and further identifies pathways that regulate the gene’s function.

ts/cs mutations often disorder protein structure or protein-protein interactions in a protein complex, and suppressors of the original ts/cs mutation often occur close to the original ts/cs mutation in protein/protein complex structure. Suppressors restore structural defects caused by the original ts/cs mutation. The current method identifies ts/cs mutants with single amino acid substitutions in targeted motifs/domains, and in combination with suppressor screening, identifies amino acid sequences (either in the same protein or in different proteins in the same complex) interacting with the original ts/cs mutation in the 3D structure. In cohesin, the N-terminal HTH motif (Helix-Turn-Helix) of Rad21 interacts with the Psm3 head-coiled coil junction (Gligoris *et al.* 2014; Huis in ’t Veld *et al.* 2014). Suppressors of *rad21-K1* (containing an I67F substitution in the Rad21 N-terminal HTH motif) were identified in the Psm3 head-coiled coil junction (Xu *et al.* 2018a) that interacts with the N-terminal HTH motif of Rad21. Most intragenic suppressors of *mis4-242* (containing a G1326E substitution at its C-terminus) were located in two regions (AA: 642∼878 in the Mis4 middle region and AA: 1316∼1415 at the Mis4 C-terminus) (Xu *et al.* 2018a). The Mis4 C-terminal region (AA: 1316∼1415) is near the original ts mutation G1326E, while the Mis4 middle region (AA: 642∼878) in far from the original ts mutation G1326E in the protein sequence. Mis4 represents a hooked structure, in which the two regions are brought close in the 3D structure (Chao *et al.* 2015; Chao *et al.* 2017; Kikuchi *et al.* 2016). Overall, suppressors of ts/cs mutants reflect the 3D structure of the protein or organization of the protein complex.

The condensin (and cohesin) hinge ts mutants will be very useful to identify proteins or domains in condensin complex itself that may directly interact with hinge domain. Head and hinge of cohesin, which are far in planar, were proposed to interact in a ‘hold and release’ model (Xu *et al.* 2018a), if suppressor mutations of these condensin and cohesin ts/cs mutants can be identified and mapped in head domain or head-associating non-SMC subunits, it will be a strong evidence to support the ‘hold and release’ model.
